# Sodium Sulfite Exacerbates Allograft Vasculopathy and Affects Tryptophan Breakdown in Murine Heterotopic Aortic Transplantation

**DOI:** 10.1155/2019/8461048

**Published:** 2019-04-08

**Authors:** Robert Sucher, Theresa Hautz, Elisabeth Mohr, Maximilian Mackowitz, Vanessa Mellitzer, Christina Steger, Benno Cardini, Thomas Resch, Christian Margreiter, Stefan Schneeberger, Gerald Brandacher, Dietmar Fuchs, Rupert Oberhuber, Johanna M. Gostner

**Affiliations:** ^1^Department of Visceral, Transplant and Thoracic Surgery, Center of Operative Medicine, Medical University of Innsbruck, Anichstr. 35, A-6020 Innsbruck, Austria; ^2^Department of Visceral, Transplant, Thoracic and Vascular Surgery, University Hospital Leipzig, Leipzig, Germany; ^3^Institute of Pathology, Academic Teaching Hospital Feldkirch, Carinagasse 47, A-6807 Feldkirch, Austria; ^4^Department of Plastic and Reconstructive Surgery, Vascularized Composite Allotransplantation (VCA) Laboratory, Johns Hopkins University School of Medicine, 733 North Broadway, Baltimore, Maryland, USA; ^5^Division of Biological Chemistry, Biocenter, Medical University of Innsbruck, Innrain, A-6020 Innsbruck, Austria; ^6^Division of Medical Biochemistry, Biocenter, Medical University of Innsbruck, Innrain, A-6020 Innsbruck, Austria

## Abstract

Graft vasculopathy is the main feature of chronic rejection in organ transplantation, with oxidative stress being a major trigger. Inflammation-associated prooxidant processes may be controlled by antioxidants; however, interference with redox-regulated mechanisms is a complex endeavor. An essential feature of the cellular immune response is the acceleration of tryptophan (Trp) breakdown, leading to the formation of several bioactive catabolites. Long-term activation of this immunobiochemical pathway contributes to the establishment of a tolerogenic environment, thereby supporting allograft survival. Herein, the impact of the antioxidant sodium sulfite on the development of graft vasculopathy was assessed in murine aortic transplantation. Allogeneic (BALB/c to C57BL/6) heterotopic murine aortic transplantations were performed. Animals were left untreated or were treated with 10 *μ*l of 0.1 M, of 0.01 M sodium sulfite, or of 0.1 M sodium sulfate, intraperitoneally once/day, until postoperative day (POD) 100. Grafts were assessed by histology, immunohistochemistry, and adhesion molecule gene expression. Serum concentrations of tryptophan and its catabolite kynurenine (Kyn) were measured. On day 100, graft vasculopathy was significantly increased upon treatment with 0.1 M sodium sulfite, compared to allogeneic untreated controls (*p* = 0.004), which correlated with a significant increase of *α*-smooth-muscle-actin, Vcam-1, and P-selectin. Serum Kyn concentrations increased in the allogeneic control group over time (*p* < 0.05, POD ≥ 50), while low-dose sodium sulfite treatment (0.01 M) treatment resulted in a decrease in Kyn levels over time (*p* < 0.05, POD ≥ 10), compared to the respective baselines (*p* < 0.05). Longitudinal analysis of serum metabolite concentrations in the different treatment groups further identified an overall effect of sodium sulfite on Kyn concentrations. Antioxidative treatment may result in ambivalent consequences. Our data reveal that an excess of antioxidants like sodium sulfite can aggravate allograft vasculopathy, which further highlights the challenges associated with interventions that interfere with the complex interplay of redox-regulated inflammatory processes.

## 1. Introduction

The incidence of acute allograft rejection has been reduced effectively by the introduction of modern immunosuppressive regimes, yet the development of chronic rejection remains a vital and unresolved issue in solid organ transplantation. The main pathophysiologic feature of chronic rejection is transplant vasculopathy with neointimal proliferation leading to vascular luminal narrowing, hypoperfusion, and fibrosis and ultimately to chronic transplant dysfunction [1, 2]. Oxidative as well as antioxidative pathways are involved in various immune and inflammatory reactions and may play a role in the development of transplant vasculopathy [3]. As for heart transplant recipients, an increase in oxidative stress and antioxidant enzyme activity was shown to be associated with development of cardiac allograft vasculopathy [4].

Inflammation-associated oxidative stress may harm tissue and trigger immune reactions; however, long-term activation of immunoregulatory pathways also contributes to the establishment of a tolerogenic environment [5], thereby supporting allograft survival. The acceleration of tryptophan (Trp) breakdown along the kynurenine (Kyn) axis is considered to be an important metabolic pathway critically involved in immunoregulation. The inflammation-associated activation of indoleamine 2,3-dioxygenase (IDO-1) accelerates the Trp breakdown rate, indicated by an increase in the Kyn/Trp ratio [6]. Kyn can be converted to several bioactive metabolites, depending on the enzymatic repertoire of the cell [7, 8]. The deprivation of Trp and the presence of some of its downstream catabolites contribute to immunoregulation by suppressing adaptive T cell-mediated responses [9]. This contributes to tolerance induction in pregnancy, autoimmunity, tumor surveillance, and transplantation [10–12]. Moreover, the hepatic enzyme tryptophan 2,3-dioxygenase (TDO) can degrade Trp, though this enzyme is mainly involved in the regulation of physiological Trp concentrations [13].

Findings of high intragraft IDO-1 levels in spontaneously tolerant murine liver and renal allografts and abrogation of tolerance upon pharmacological inhibition of IDO-1 activity underline the allograft protective capacity of this metabolic pathway [14, 15]. In addition, *Ido1* gene therapy supported the prevention of acute rejection of the skin [16], heart [17], lung [18], kidney [19], and pancreatic islet allograft rejection [20]. However, the role of Trp catabolism in allograft rejection is two-sided: chronic accelerated tryptophan breakdown may support the maintenance of a tolerogenic environment due to metabolic control of the immune response, but this may also promote growth of malignant cells [21]. This immunobiochemical pathway is activated parallel to host defense mechanisms directed also against non-self-tissue [22]. In addition, several Trp catabolites may affect plasma membrane fluidity [23]. Thus, the physiologic role of tryptophan breakdown in solid organ transplantation is not yet completely understood.

Oxidative stress management during organ transplantation is crucial for a positive outcome. A number of antioxidant treatments are under investigation, for the donor, during graft preservation, or for the recipient [24]. However, interfering with redox regulation may be challenging. *In vitro* experiments with human peripheral blood mononuclear cells showed that antioxidants including sodium sulfite significantly suppress mitogen-induced IDO-1 activity in a dose-dependent manner and hence attenuated immune activation [25–27]. Sodium sulfite has been used as a food preservative since ancient times. Still today, sulfites and sulfiting agents are utilized for cosmetic and medical purposes due to their purifying and disinfectant properties [28].

In this study, a model of murine heterotopic aortic transplantation was applied to investigate the impact of sodium sulfite on the development of graft vasculopathy. In addition to the assessment of the final outcome, serum concentrations of biomarkers Trp and Kyn were determined at different time points after transplantation.

## 2. Materials and Methods

### 2.1. Animals

Male C57BL/6 (H-2b) and BALB/c (H-2d) mice (8-12 weeks) weighing 20-25 g were obtained from Charles River Laboratories (Sulzfeld, Germany). Animals were housed under standard conditions and given mouse chow and water *ad libitum* before and after transplantation. Animals received humane care in compliance with the “Principles of Laboratory animal care” (NIH Publication Vol. 25, No. 28 revised 1996) as well as with specific national laws. All experiments were approved by the Austrian Ministry of Education, Science and Culture (BMWF-66011/0038-II/3b/2011).

### 2.2. Murine Heterotopic Aortic Transplantation

Anaesthesia was performed via an intramuscular injection of 100 mg/kg ketamine hydrochloride (Ketasol®, Dr. E. Graeub, Switzerland) and 10-15 mg/kg xylazine (Xylasol®, Dr. E. Graeub, Switzerland), respectively. Intra- and postoperative analgesia was achieved by 4 mg/kg carprofen (Rimadyl®, Pfizer, Austria), injected subcutaneously once a day until postoperative day (POD) 4.

The donors' thoracic aorta was retrieved and transplanted as a carotid interposition graft after 12 hours of cold ischemia time (CIT = total time on ice at 4°C). The degree of ischemia/reperfusion injury influences the severity of transplant vasculopathy [29], and 12 hours of CIT have been shown to induce slowly progressing vascular hyperplastic lesions in this major histocompatibility complex-mismatched model [30]. The preservation solution used in the present study, Custodiol® (HTK, Dr. Franz Köhler Chemie GmbH, Germany) is composed of electrolytes at concentrations similar to an intracellular concentration (sodium, calcium, potassium, and magnesium) and further contains an amino acid buffering agent, histidine/histidine hydrochloride, Trp, a-ketoglutarate, and the osmotic agent mannitol. Vascular anastomoses were performed using a cuff technique as previously described [29]. Thereby, both ends of the donor aorta were subsequently everted over and fixed on polyethylene cuffs (outer diameter 0.63 mm, Portex Ltd., UK). Warm ischemia time (= time from removal from ice to reperfusion of the graft) was kept at a minimum (approximately 5 minutes).

### 2.3. Experimental Design

In total, five groups (*n* = 8 animals/group) were included, and animals were treated as follows: group 1: syngeneic control group (BALB/c to BALB/c mice), group 2: allogeneic group control group (BALB/c to C57BL/6), group 3: allogeneic high-dose treatment group—recipient animals were treated with a daily injection of 100 *μ*l of 0.1 M sodium sulfite (Merck, Germany) applied intraperitoneally (i.p.), group 4: allogeneic low-dose treatment group—recipients were treated with a daily injection of 100 *μ*l of 0.01 M sodium sulfite (Merck, Germany) i.p., and group 5: allogeneic recipients treated with a daily injection of 100 *μ*l of 0.1 M sodium sulfate (Merck, Germany). Sodium sulfate treatment was performed as internal control, due to the relative nonreactivity of sodium sulfate in aqueous solutions compared to sodium sulfite. Treatment was started right after the transplantation procedure and continued once/day until POD 100, when aortic grafts and serum samples were collected for further investigation. Additional serum samples were collected from the tail vein on POD 0, 5, 10, 15, 50, and 75.

### 2.4. Histopathology

Aortic grafts were fixed in 4% buffered formaldehyde and embedded in paraffin. For morphological evaluation, three sections per aortic graft (4 *μ*m) were stained with hematoxylin and eosin (H&E), as well as Elastica van Gieson, according to standard procedures. Vessels were photographed at 10- and 20-fold magnification with a digital camera (Canon PowerShot G5, Austria). Each section was divided into quadrants, each of which was evaluated for intima and media thickness. The mean was calculated from three cross sections per animal. Analyses were carried out using ImageJ 1.32j software for Java (National Institutes of Health, USA).

### 2.5. Immunohistochemistry (IHC)

To analyse the number of smooth muscle cells in the intima of aortic grafts, IHC was performed on paraffin-embedded sections, using an anti-alpha-smooth muscle actin (*α*-SMA) antibody (Clone 1A4, Code M0851, dilution 1 : 100, Dako, Denmark) in accordance with the manufacturer's instructions [31] and the Vector DAB substrate kit for peroxidase (SK-4100, Vector Laboratories Inc., USA). Counterstaining war performed with hematoxylin. Immunoreactivity against *α*-SMA was scored semiquantitatively in a blinded fashion as follows: 0—no immunostaining, 1—slight immunostaining, few cells positive, 2—mild positive staining, cells in clusters over the whole circumference, and 3—intense immunostaining over the whole circumference.

### 2.6. Trp and Kyn Measurements

Serum Trp and Kyn concentrations were measured by reversed-phase high-pressure liquid chromatography on a ProStar Varian system, as described earlier [32, 33]. In brief, chromatographic separation was performed using a RP-18 column (Merck, Germany) and 15 mmol/l acid-sodium acetate buffer (pH 4.0) as eluent (flow rate: 0.9 ml/min). Serum specimens were deproteinized with trichloroacetic acid (2 mol/l) before analysis. 3-Nitro-L-tyrosine was used as an internal standard. All chemicals were purchased from Sigma-Aldrich (Austria). Trp was monitored by its native fluorescence at 286 nm excitation and 366 nm emission wavelengths (ProStar 360 detector, Varian, USA); Kyn and 3-nitro-L-tyrosine were detected by UV absorbance at the 360 nm wavelength (Shimadzu SPD-6A UV detector, Austria) in the same chromatographic run. Finally, the Kyn to Trp ratio (Kyn/Trp; kynurenine (*μ*mol/l) divided by Trp (mmol/L)) was calculated [6].

### 2.7. Quantitative Polymerase Chain Reaction (qPCR)

Total RNA from snap-frozen aortae was extracted using NucleoSpin® RNA Kit (Macherey-Nagel, Germany). cDNA was preamplified using a target-specific pooled primer mix, nuclease-free water, GeneAmp® 10x PCR Buffer (Thermo Fisher Scientific, USA), and TaqMan™ PreAmp Master Mix (Thermo Fisher Scientific, USA) adjusted to a final volume of 25 *μ*l. The reaction was carried out using the ABI PRISM 7500 Sequence Detection System (Life Technologies, Germany) with the following conditions: 95°C, 10 min, cycling (10 cycles): 15 sec denaturation step at 95°C and 4 min annealing/extension step at 60°C. Primers were either designed with Primer Express software (Life Technologies, CA, USA) and validated in-house or purchased as assays on demand (Thermo Fisher Scientific, USA; vascular cell adhesion molecule-1 (Vcam-1), assay ID: Mm01320970_m1, and P-selectin assay ID: Mm01295931_m1). The qPCR reaction was performed in a final reaction volume of 13 *μ*l containing 2.5 *μ*l preamplified cDNA, 6.5 *μ*l TaqMan™ Universal PCR Master Mix (Thermo Fisher Scientific, USA), 0.5 *μ*l of the respective primer mix, and 3.5 *μ*l nuclease-free water. Amplification consisted of a two-step qPCR (40 cycles; 15 sec denaturation step at 95°C and 1 min annealing/extension step at 60°C). The relative expression of target genes was calculated based on the normalized Ct deviation of the samples from the respective treatment group versus the control group according to the mathematical model described by Pfaffl [34]. Expression differences between groups were compared using the pairwise fixed reallocation randomization test of the REST© relative expression software tool [35].

### 2.8. Statistical Analysis

Results are expressed as mean ± standard error of the mean (SEM). Statistical analysis was performed using IBM SPSS Statistics for Windows, version 24 (IBM Corporation, USA). As not all data showed normal distribution, nonparametric methods were applied. Friedman and Wilcoxon signed-rank tests were used to analyse changes within each group over time, and Kruskal Wallis and Mann–Whitney *U* tests were used for comparisons between treatment groups. A *p* value of <0.05 was considered to be of statistical significance (n.s. = not significant).

Longitudinal data analysis was performed using a linear model with general covariance structure and analysis using restricted maximum likelihood (REML) estimation according to the protocol of Duricki et al., with minor modifications [36]. The posttreatment performance was analysed for each group, after controlling for individual differences in baseline parameters (Trp, Kyn, and Kyn/Trp). A limitation of the analysis was that only Trp data followed normal distribution at all time points within all treatment groups, as analysed with Kolmogorov-Smirnov testing, while Kyn and Kyn/Trp showed normal distribution only after subanalysis of groups or time points in most but not all conditions. Variances become somewhat smaller at later time points, and similarity of group variances could be assumed for most time points. The full dataset obtained was used for analysis, and no outlayers were defined. Linear models with alternative covariance structures and REML estimation were explored, and the <first –order autoregressive (AR1)> covariance structure had the best fit and resulted in the best Akaike's Information Criterion (Trp: -131, log(Kyn): -95). For Trp, the residuals appeared to come from a normal distribution; for Kyn residuals, *p* = 0.048 was obtained in the Kolmogorov-Smirnov analysis and normal distribution became significant after log transformation of Kyn concentrations (nM). For Kyn/Trp, the assumption that residuals come from a normal distribution could not be confirmed. Fixed factors in the final models were group, POD, and group by POD interaction. Respective baseline scores (POD = 0) were used as covariate. Bivariate correlation (Pearson's) was performed to investigate the correlation structure between Trp, Kyn, and Kyn/Trp at each time point for each group.

## 3. Results

### 3.1. Sodium Sulfite Increases Intimal Thickening in a Major Histocompatibility Complex-Mismatched Aortic Transplant Model

**Figure 1:**
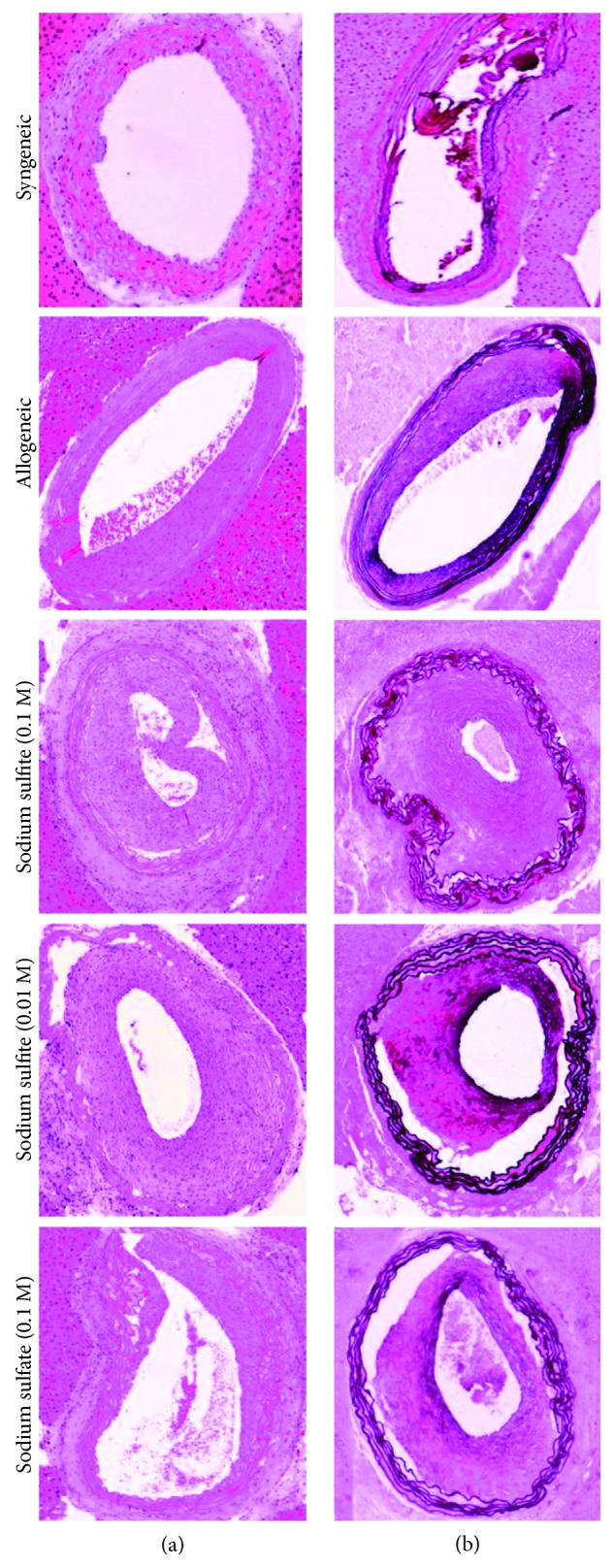
H&E (a) and Elastic van Gieson (b) staining of aortic grafts collected on postoperative day 100. Representative images are shown for each group (magnification: ×10).

To quantify the extent of graft vascular hyperplasia, intima and media thickness of grafts were assessed in H&E- and Elastic van Gieson-stained sections on POD 100. Independent of the treatment applied, all allogeneic recipients developed graft vasculopathy as evidenced by significant intimal hyperplasia leading to luminal narrowing (Table 1, Figure 1). Syngeneic grafts did not show any alterations. Of note, daily administration of high- and low-dose sodium sulfite resulted in a significant exacerbation of intimal hyperplasia (0.1 M: 129 *μ*m ± 7.00, 0.01 M: 110 *μ*m ± 6.93) compared to sodium sulfate treatment (89.5 ± 2.11; Table 1). Moreover, a significant increase in the intima/media ratio was found for the 0.1 M sodium sulfite treatment group (3.44 ± 0.17), compared to the allogeneic (2.29 ± 0.26) as well as sodium sulfate-treated (2.45 ± 0.19) recipients (Table 1). Figure 1 presents representative images of H&E- (A) and Elastic van Gieson staining (B) of each group.

### 3.2. Sodium Sulfite Enhances *α*-SMA Expression in Aortic Allografts

**Figure 2:**
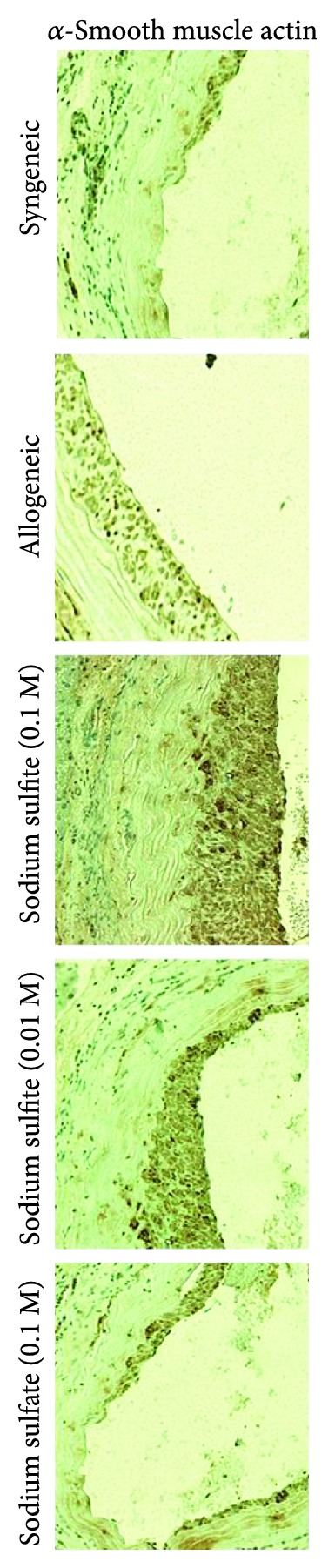
Immunohistochemical staining for *α*-SMA revealed a significant increase in positive-stained cells in the intima of all allogeneic aortic grafts on postoperative day 100, regardless of treatment given to the recipient, compared to syngeneic grafts. In the sodium sulfite (0.1 M) treatment group, a significant increase even compared to allogeneic, untreated controls was observed. Representative images are shown for each group (magnification: ×20).

In order to distinguish intimal hyperplasia from unspecific arteriosclerotic lesions, IHC staining for *α*-SMA was performed in aortic grafts on POD 100. In line with the results from the morphological assessment, IHC revealed a significant increase in *α*-SMA-positive cells within the intima of all allogeneic groups, as compared to the syngeneic grafts, which showed almost no *α*-SMA-positive cells beneath the endothelium (Figure 2). Aortic grafts from 0.1 M sodium sulfite-treated animals showed a significantly increased expression of *α*-SMA as compared to allogeneic controls (2.86 ± 0.14 vs. 1.86 ± 0.26, Table 1), but there were no such differences for grafts from low-dose 0.01 M sodium sulfite and the sodium sulfate-treated animals.

### 3.3. Serum Trp and Kyn Concentrations

**Figure 3:**
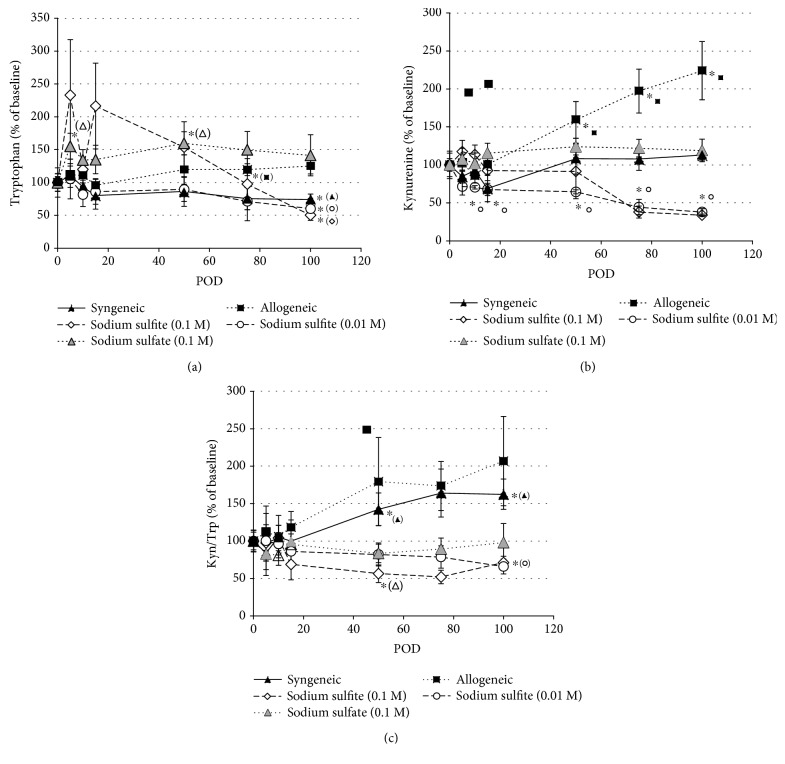
Changes in tryptophan (a) and kynurenine (b) concentrations and Kyn/Trp (c) of the individual treatment groups normalized to baseline (POD = 0) over the observation period of 100 days (POD = postoperative day). Mean values ± SEM are shown, ^∗^*p* < 0.05 indicates significance compared to baseline.

The concentration of Trp and its first stable metabolite Kyn were analysed in the serum of recipient animals on POD 0, 5, 10, 15, 50, 75, and 100. At baseline, the mean Trp concentration (± SEM) was 66.9 ± 4.83 *μ*mol/l, the mean Kyn level was 1.30 ± 0.07, and the mean Kyn/Trp was recorded to be 21.9 ± 1.46 (*n* = 33). The baseline concentrations varied between the syngeneic and allogeneic recipient groups, but differences were not significant among the allogeneic groups (Table 2). For comparison of posttreatment concentrations, data were normalized to baseline (POD = 0). The time course of changes in the concentrations is shown in Figures 3(a) and 3(b). Kyn/Trp indicates the Trp breakdown rate (Figure 3(c)).

In syngeneic transplanted animals, Trp concentrations slightly declined over time, however, reaching significance only on POD 100 with a decline of 26%, compared to baseline (POD = 0). Kyn concentrations showed a decrease during the first 15 days. From POD 50 to 100, Kyn concentrations increased compared to baseline, though the effect was not significant. However, Kyn/Trp increased significantly starting from POD 50 on and resulting in a 1.6-fold higher Kyn/Trp on POD 100 as compared to baseline (Figure 3(c)).

In allograft-recipient animals, Trp and Kyn concentrations were significantly increased compared to baseline on days 15 and 50 to 100, respectively. Kyn concentrations were more than 2-fold elevated on POD 100. Kyn/Trp showed the same trend; however, changes failed to be significant.

In 0.1 M sodium sulfite-treated allogeneic recipients, compared to baseline, a nonsignificant increase in Trp concentrations was observed until POD 50 followed by a significant decrease at later time points. Kyn concentrations showed analogous but less pronounced trends. Kyn/Trp was decreased from POD 15 onwards, becoming significant only on POD 50, when Kyn/Trp was approximately half of baseline Kyn/Trp. In the group receiving 0.01 M sodium sulfite, Trp concentrations slightly decreased from POD 10 onwards, reaching a significant 40% decrease on POD 100. Kyn concentrations showed a significant reduction compared to baseline from POD 10 to 100. A trend towards a decline of Kyn/Trp was observed, resulting in a 35% decrease compared to baseline on POD 100.

In the sodium sulfate treatment control group, Trp concentrations increased on POD 5 and remained elevated (1.5-fold) compared to baseline over the whole treatment period; however, this effect was significant at PODs 5 and 50 only. Kyn concentrations were somewhat increased, and Kyn/Trp slightly decreased, but there was no significant difference compared to baseline over the whole observation period.

**Figure 4:**
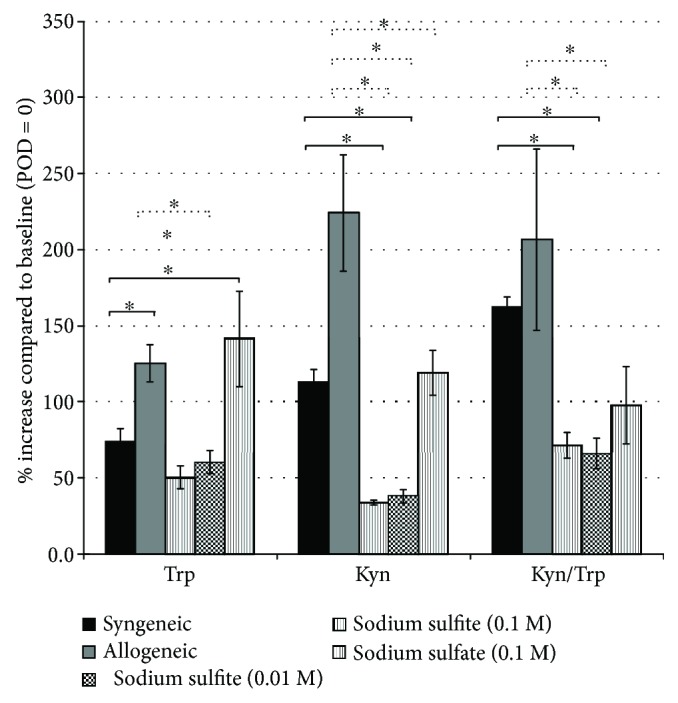
Comparison of tryptophan and kynurenine concentrations and Kyn/Trp on POD 100 (normalized to POD = 0, set as 100%). Mean values ± SEM are shown; ^∗^*p* < 0.05 indicates significant difference compared to the syngeneic (continuous line) or allogeneic group (broken line), respectively. For the sake of clarity, significant differences between the other treatment groups are not indicated.

The changes in Trp and Kyn levels and of Kyn/Trp compared to baseline on POD 100 are depicted in Figure 4. Concentrations were normalized to baseline (POD 0 = 100%). On POD 100, Trp levels were decreased in both sodium sulfite treatment groups compared to the allogeneic control group, but not compared to the sodium sulfate control group. Likewise, Kyn concentrations were significantly decreased in the high- and low-dose sodium sulfite treatment group and in sodium sulfate animals, compared to allogeneic controls. Kyn/Trp was significantly deceased in both sodium sulfite groups as compared to the allogeneic untreated controls, indicating a decreased Trp breakdown rate. Though a similar trend was observed in the sodium sulfate-treated animals, this was not significant.

**Figure 5:**
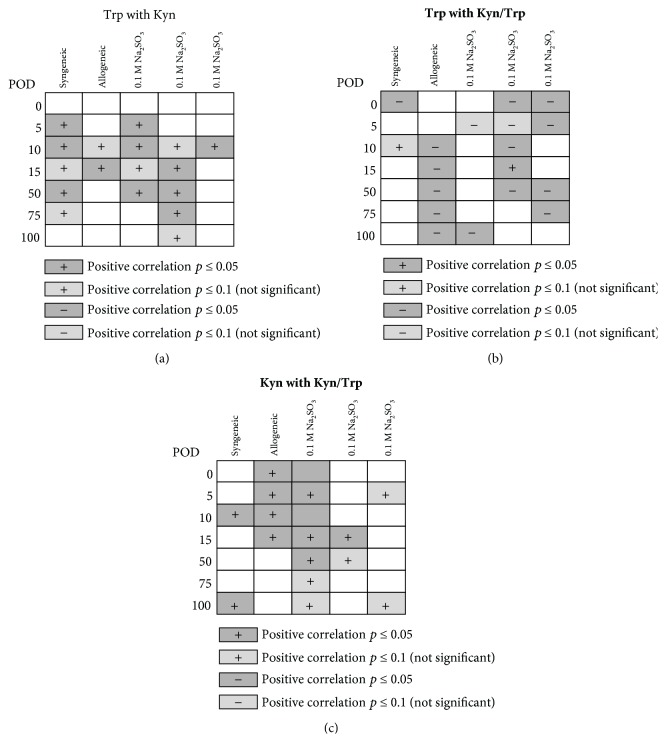
Pearson's correlations were calculated for the biomarker concentrations at individual postoperative days (POD) for each group: Trp with Kyn (a), Trp with Kyn/Trp (b), and Kyn with Kyn/Trp (c). Significant correlations (*p* ≤ 0.05) are indicated in grey, and light grey indicates correlations with a *p* value ≤0.1.

Correlation analysis of these biomarkers at individual time points and within individual treatment groups revealed a positive correlation of Trp and Kyn concentrations for several time points (Figure 5(a)). As expected, inverse correlations of Trp concentrations with Kyn/Trp were observed, while Kyn and Kyn/Trp correlated positively to each other (Figures 5(b) and 5(c)).

A REML model was applied to assess the longitudinal effects of the different treatments on Trp, Kyn, and Kyn/Trp concentrations. Exploring the fixed effects after controlling for baseline differences of Trp concentrations, there was neither a significant effect of group, postoperative time (POD), nor interaction of group and time. After controlling for baseline differences of the biomarker concentrations, for Kyn (log Kyn) there was an effect of group (*F*_4,43.219_ = 19.728, *p* ≤ 0.001) and an interaction of group with time (*F*_20,106.110_ = 3.982, *p* ≤ 0.001), but not of postoperative time alone. Pairwise comparisons of group means showed a difference between sodium sulfite-treated animals (both concentration) and syngeneic as well as allogeneic animals. This difference was also significant when comparing sulfite-treated animals and the sodium sulfate group.

### 3.4. Expression of Vcam-1 and P-Selectin on mRNA Levels in Aortic Grafts

In order to identify potential consequences on the expression of cell adhesion molecules, Vcam-1 and P-selectin mRNA levels were measured by qPCR in aortic grafts collected on POD 100. Compared to syngeneic controls, Vcam-1-specific mRNA expression was significantly upregulated in all allogeneic recipients, while P-selectin was found to be significantly increased in untreated and high-dose (0.1 M) sodium sulfite-treated animals of the allogeneic groups. When comparing the relative expression levels with the allogeneic grafts, the 0.1 M sodium sulfite group revealed a significant upregulation of both Vcam-1 (*p* < 0.001) and P-selectin (*p* = 0.001) mRNA (Table 3).

## 4. Discussion

Chronic allograft rejection remains a major obstacle when improving long-term outcome after solid organ transplantation. Chronic transplant dysfunction has been identified as the main cause for long-term graft loss [1, 37], mostly attributable to the development of transplant vasculopathy with intimal hyperplasia and luminal narrowing of allograft vessels, consequently leading to chronic hypoperfusion of the graft and interstitial fibrosis [38].

In this study, allogeneic recipient animals developed accelerated graft vasculopathy, as evidenced by an increased intima/media ratio, when treated with 100 *μ*l of 0.1 M sodium sulfite for 100 days, as compared to syngeneic, allogeneic untreated, and sodium sulfate-treated controls in a murine heterotopic aortic transplant model. In addition, compared to the syngeneic and allogeneic control group, both Kyn concentrations and Kyn/Trp decreased upon sodium sulfite treatment, showing somewhat more effects with 0.01 and 0.1 M treatment. An overall effect of sodium sulfite treatment was also identified in the longitudinal data analysis.

Results of the correlation analysis of Trp and Kyn with Kyn/Trp for each group at each time point indicate ongoing catabolism of Trp along the Kyn axis. Interestingly, for several time points, a positive correlation of Trp and Kyn concentrations was found. A better nutritional status due to the recovery of the animal from transplantation could explain elevated Trp levels, leading also to some increase of kynurenine. The hepatic enzyme TDO is responsible for the regulation of Trp concentrations in the circulation. However, this competes with the accelerated breakdown of tryptophan due to inflammation-associated induction of IDO-1, finally leading to a pathological increase of kynurenine.

Larkin et al. reported that Kyn levels in *Ido1^−/−^* mice tended to be somewhat lower compared to *Ido1^+/+^*, but the differences in concentrations did not reach statistical significance [36]. Therefore, we cannot rule out a contribution of other enzymes as IDO-1 to reduce Trp levels in the sodium sulfite-treated animals. The Kyn/Trp ratio can only serve as an estimate of IDO-1 activity, if it correlates with another marker of immune activation [6, 26].

Recently, it was shown that *Ido1* gene therapy protects against the development of transplant vasculopathy in a rat model of chronic kidney transplant dysfunction [39]. By employing adenovirus-delivered *Ido1* gene therapy in renal allografts, the authors showed a beneficial effect on transplant vasculopathy in a rat model of renal chronic transplant dysfunction. The concept of IDO-1 acting as an inhibitor of graft arteriosclerosis has also been suggested by Cuffy et al. [40]. Induction of IDO-1 activity in human vascular smooth muscle cells by interferon-*γ* inhibited allogeneic T cell activation, proliferation, and accumulation, whereas IDO-1 inhibition by 1-methyl-tryptophan increased artery-infiltrating T cells, which may contribute to the development of atherosclerosis and graft arteriosclerosis.

Several compounds were shown to inhibit IDO-1 activity *in vitro*, such as antioxidants [27]. In this context, the effect of frequently used reducing agents in food, chemical, cosmetic, and pharmaceutical industry was of interest, specifically focusing on the preservative sodium sulfite [28]. In the present *in vivo* study, sodium sulfite was applied as a model compound for a preservative antioxidant, due to its dose-dependent suppressive effect on IDO-1 activity reported in an *in vitro* study with human peripheral blood mononuclear cells [26]. Sodium sulfate served as a control compound due to its relative nonreactivity compared to sodium sulfite.

In addition to the effects on Trp breakdown, enhanced endothelial cell activation as evidenced by significantly increased mRNA levels of Vcam-1 and P-selectin in aortic grafts after high-dose (0.1 M) sodium sulfite treatment may have contributed to the exacerbation of aortic graft vasculopathy. Both adhesion molecules are highly upregulated by endothelial cells after cytokine stimulation during immune activation [41, 42]. They mediate adhesion of leukocytes to vascular endothelium, allowing them to transmigrate through the vessel wall. Thus, it can be hypothesized that a higher number of immunocompetent cells were recruited in these allografts, resulting in a chronic state of inflammation of the vascular layers, ultimately and in conjunction with IDO-1 inhibition leading to enhanced intima hyperplasia. Decreased IDO-1 activity itself may have contributed to increased immune cell infiltration after sodium sulfite treatment, as IDO-1 has been shown to reduce cell infiltrates [43]. Unfortunately, the heterotopic aortic transplant model is not the ideal model to quantify and characterize the infiltrating immune cells, as the allograft itself is relatively small and allograft stroma is short.

Finally, interference of Trp metabolism might have had an impact on blood pressure as Kyn is suggested to be involved in blood pressure lowering under inflammatory conditions by inducing the relaxation of blood vessels [44, 45]. This may act as an additional stressor for endothelial activation.

## 5. Conclusion

This study demonstrates that the sodium sulfite exaggerates graft vasculopathy in a murine major histocompatibility complex-mismatched aortic transplant mode, whereby the interference with Trp catabolism may provide a mechanistic explanation. The suppression of Trp breakdown as previously reported *in vitro* could be shown *in vivo*, too. Thus, antioxidant treatments, which are generally considered as anti-inflammatory, may result in ambivalent consequences. Due to the multifaceted role of immunoregulatory pathways and the importance of temporal regulation, further studies are warranted to explore these observations in detail.

## Figures and Tables

**Table 1:** Intima thickness, intima to media ratio, and immunohistochemical *α*-smooth muscle actin on POD 100.

	Syngeneic	Allogeneic	Sodium sulfite (0.1 M)	Sodium sulfite (0.01 M)	Sodium sulfate (0.1 M)
Mean intima thickness ± SEM (*μ*m)	7.36 ± 3.43	96.1 ± 11.0^∗^	129±7.00^∗,a^	110±6.93^∗,a^	89.5 ± 2.11^∗^
Mean intima to media ratio ± SEM	0.16 ± 0.08	2.29 ± 0.26^∗^	3.44 ± 0.17^∗^^,#,a^	2.68 ± 0.27^∗^	2.45 ± 0.19^∗^
*α*-Smooth muscle actin semiquantitative score	0.43 ± 0.20	1.86 ± 0.26^∗^	2.86±0.14^∗,^^#,a,b^	2.00 ± 0.32^∗^	1.80 ± 0.20^∗^

Significant differences (*p* < 0.05): ^∗^compared to the syngeneic group, ^#^compared to the untreated allogeneic group, ^a^compared to the sodium sulfate group, and ^b^compared to the 0.01 M sodium sulfite treatment group.

**Table 2:** Baseline concentrations (POD = 0) of tryptophan, kynurenine, and the Kyn/Trp ratio of all groups are shown (mean ± SEM, *n* ≥ 5).

	Tryptophan (*μ*mol/l)	Kynurenine (*μ*mol/l)	Kyn/Trp (mmol/*μ*mol)
Syngeneic	95.6 ± 12.6	1.59 ± 0.09	18.0 ± 2.1
Allogeneic	56.6 ± 4.6^∗^	1.07 ± 0.18	19.8 ± 3.1
Sodium sulfite (0.1 M)	59.1 ± 13.2	1.14 ± 0.21	21.1 ± 3.0
Sodium sulfite (0.01 M)	77.3 ± 11.5^∗^	1.31 ± 0.02^∗^	18.7 ± 2.9
Sodium sulfate (0.1 M)	54.4 ± 4.6^∗^	1.46 ± 0.11	28.5 ± 3.9

Significant differences (*p* < 0.05): ^∗^compared to syngeneic.

**Table 3:** mRNA expression of Vcam-1 and P-selectin in aortic grafts in comparison to syngeneic controls (upper rows) and in comparison to allogeneic, untreated controls (lower rows).

	Syngeneic	Allogeneic	Sodium sulfite (0.1 M)	Sodium sulfite (0.01 M)	Sodium sulfate (0.1 M)
Relative expression compared to syngeneic recipients					
*Vcam-1*					
Relative exp.	**1**	**2.46**	**7.11**	**2.91**	**2.54**
SD		1.18–4.74	3.61–14.0	1.61–6.07	1.51–4.71
95% C.I.		0.52–9.71	1.66–26.8	0.70–9.16	0.70–6.96
p(H1)		*0.012*	*0.001*	*0.003*	*0.004*
P-selectin					
Relative exp.	**1**	**1.63**	**3.83**	**1.57**	**1.05**
SD		1.15–2.32	2.22–6.84	0.76–2. 90	0.41–2.20
95% C.I.		0.85–3.26	1.69–10.1	0.45–5.23	0.14–2.71
p(H1)		*0.005*	*0.002*	0.109	0.900

Relative expression compared to allogeneic recipients					
*Vcam-1*					
relative exp.		**1**	**2.89**	**1.19**	**1.04**
SD			1.69–4.62	0.73–1.85	0.66–1.49
95% C.I.			1.36–6.47	0.51–3.21	0.57–1.94
p(H1)			*<0.001*	0.403	0.820
P-selectin					
Relative exp.		**1**	**2.35**	**0.96**	**0.65**
SD			1.39–4.28	0.47–1.62	0.27–1.30
95% C.I.			0.93–5.92	0.32–3.15	0.10–1.58
p(H1)			*0.001*	0.877	0.272

SD = standard deviation; C.I. = confidence interval; p(H1) = hypothesis test *p* value, significant if <0.05, *n* = 7.

## Data Availability

The data used to support the findings of this study are available from the corresponding author upon request.
